# COVID-19 impact on familial relationships and mental health in a representative sample of adults: Giacomo Pietro Vigezzi

**DOI:** 10.1093/eurpub/ckac130.181

**Published:** 2022-10-25

**Authors:** GP Vigezzi, M Zeduri, G Carioli, A Lugo, A Amerio, G Gorini, R Pacifici, P Politi, S Gallus, A Odone

**Affiliations:** 1 Department of Public Health, University of Pavia, Pavia, Italy; 2 Ca’ della Paglia College, Ghislieri Foundation, Pavia, Italy; 3 Department of Community Health, University of Milan, Milan, Italy; 4 Department of Environmental Health Sciences, IIRCCS Mario Negri Institute for Pharmacological Research, Milan, Italy; 5 DINOGMI, University of Genoa, Genoa, Italy; 6 IRCCS San Martino Polyclinic Hospital, Genoa, Italy; 7 Oncologic Network, Prevention and Research Institute, Firenze, Italy; 8 National Centre on Addiction and Doping, Italian National Institute of Health, Rome, Italy; 9 Department of Brain and Behavioral Sciences, University of Pavia, Pavia, Italy

## Abstract

**Background:**

Benefits of the stay-at-home order imposed in Italy to prevent SARS-CoV-2 transmission need to be weighed against its impact on citizens’ health. In a country with a solid familial culture and where welfare relies on households, confinement drastically decreased support provided by elder relatives, which may have worsened mental health.

**Methods:**

A web-based cross-sectional study (LOST in Italy) was conducted on a representative sample of Italian adults during lockdown (27th of April-3rd of May 2020). We asked 3156 subjects to report on reduced help in housework and childcare from retired parents to assess confinement impact on mental health through validated scales before and during the lockdown.

**Results:**

Overall, 1484 (47.0%) subjects reported reduced housework help from parents, and 769 (64.0%, of the 1202 subjects with children) diminished babysitting support. Subjects reporting reduced housework help had worsened sleep quality (multivariate odds ratio, OR 1.74, 95% confidence interval, CI 1.49-2.03) and quantity (OR 1.50, 95%CI 1.28-1.76), depressive (OR 1.32, 95% CI 1.14-1.53) and anxiety symptoms (OR 1.53, 95%CI 1.32-1.78), compared to those reporting unreduced help. Worsening in sleep quality (OR 2.32, 95%CI 1.76-3.05) and quantity (OR 1.80, 95%CI 1.36-2.37), depressive (OR 1.79, 95%CI 1.39-2.31) and anxiety symptoms (OR 1.90, 95%CI 1.48-2.46) was also associated with reduced babysitting help. In subjects with poorer housing and teleworking, mental health outcomes were worse.

**Conclusions:**

Confinement came along with reduced familial support from parents, negatively impacting mental health. Social networks and support within families provided by older relatives act as a resilience factor and a potential vulnerability that affects mental health outcomes. Health and social services response should be designed to address mental health needs and mitigate long-term health costs caused by the pandemic's unprecedented stressfulness and unknown duration.

**Key messages:**

- National lockdown measures came along with reduced housework help supply for a large proportion of adult parents who presented increased mental health symptoms with unsatisfactory quality of life.
- A global, multi-level socioeconomic interdisciplinary approach is needed to inform evidence-based family and welfare policies and prevention strategies centred on population wellbeing.

